# Comparison of insertion time, pull-out strength, and screw-media interface area of customized pedicle screw with different core and thread design against commercial pedicle screw: a pilot study on Indonesian Population

**DOI:** 10.1186/s13104-021-05803-5

**Published:** 2022-01-12

**Authors:** Rahadyan Magetsari, Tedjo Rukmoyo, Marda Ade Saputra, Yudha Mathan Sakti

**Affiliations:** 1 Department of Orthopaedics and Traumatology, Dr. Sardjito General Hospital/Faculty of Medicine, Public Health, and Nursing, Universitas Gadjah Mada, Yogyakarta, Indonesia; 2 Orthopaedic and Traumatology Division, Department of Surgery, Faculty of Medicine, Universitas Gadjah Mada/Dr, Sardjito Hospital, Jl. Kesehatan No. 1, Yogyakarta, 55281 Indonesia

**Keywords:** Pedicle screw, Pull-out strength, Insertion time, Interface area

## Abstract

**Objective:**

This research aimed to developing customized pedicle screw based on Indonesian vertebral anatomy and compare the insertion time, pull-out strength, and screw-media interface area of different screw design. We have developed 3 different types of pedicle screws (v-thread cylinder-core, square-thread cylinder-core and square-thread conical-core). The thread diameter was calculated from pedicle width of Indonesian population (6 mm). We used commercially available pedicle screw as control group (6.2 mm).

**Result:**

The insertion time were significantly difference between v-thread cylinder-core pedicle screw (22.94 s) with commercially available pedicle screw (15.86 s) (p < 0.05). The pull-out strength was significantly difference between commercially available pedicle screw (408.60 N) with square-thread conical pedicle screw (836.60 N) (p < 0.05). The square-thread conical-core group have the highest interface area (1486.21 mm^2^). The data comparison showed that the square-thread conical-core customized pedicle screw group has comparable insertion time and has better pull-out strength than commercially available pedicle screw.

## Introduction

Pedicle screw is widely used in spinal surgeries. It is as a gold standard for fusion procedure of spine. The example uses of pedicle screw are in correcting scoliosis deformity, disc degenerative disease, infection in spine, tumor, and fractures [1].

In Indonesia, the use of pedicle screw is still limited due to its cost and complexity in its instruments for application. There is no single factory in Indonesia that has been able to produce pedicle screw. The needs for pedicle screws are all being full filled by importing from aboard.

Although pedicle screw has the advantage as one of the most rigid fixator [2], failure of fixation is still can occur. Factor that can be one of potential risk for failure of fixation is pull-out strength of pedicle screw. Some studies have reported that different design of thread and core of pedicle screw affect its biomechanical properties like insertion time and pull-out strength [3–5]. Size of the screw is also important, with bigger size and larger surface area correlates with greater pull-out strength, but it is also limited by the size of the anatomical size of the pedicle. Study of anatomical size of pedicle in Indonesian population that had been held by Wibowo and Suwardi in 2017, found that it has smaller size compare to western population where commercially available pedicle screws are being produced. A study conducted based on Indonesian population has observed that the mean diameter of Indonesian pedicle is 6.48 mm [6, 7]. In this case, the challenge is to produce pedicle screws with specific size based on Indonesian pedicle anatomy and designs that have comparable insertion time and pull-out strength to commercially available pedicle screws.

The aim of this study was to develop a customized pedicle screw based on Indonesian vertebral anatomy and compare the insertion time, pull-out strength, and screw-media interface area of different screw design.

## Main text

### Material and methods

**Fig. 1:**
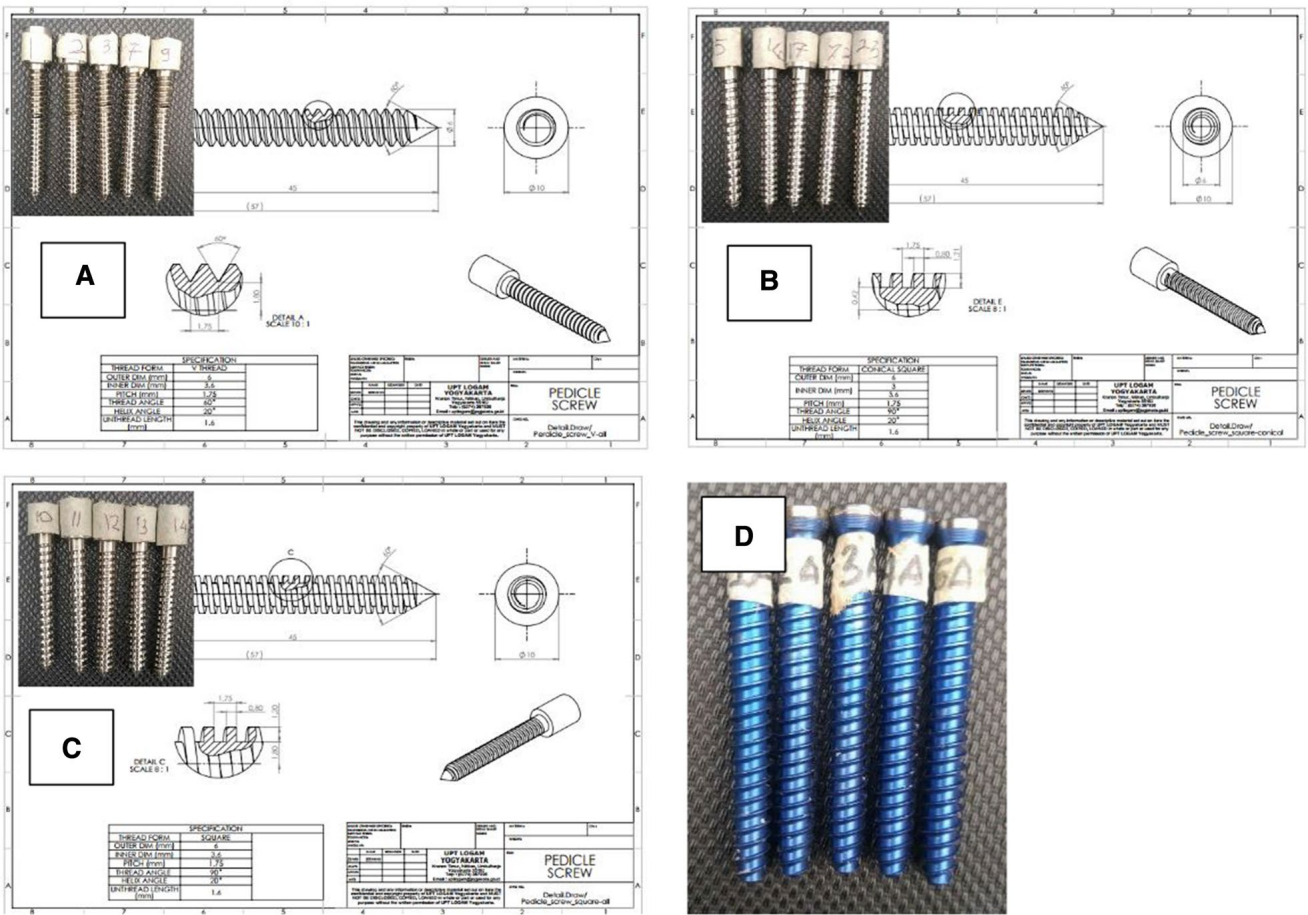
Design and final result of customized screw; **A** Cylinder core, **B** Square-thread cylinder-core and **C** Square-thread conical-core and commercially available pedicle screw (**D**) EXPEDIUM® 5,5 Spine System pedicle screw (DePuy Synthes, United States, California)

This was an experimental study. We used 4 groups of pedicle screws with 5 screws each, that consist of group A (v-thread cylinder-core pedicle screw), group B (square-thread conical-core pedicle screw), group C (square-thread cylinder-core pedicle screw), and group D (commercially available pedicle screw). We used 6 mm of diameter for our customized pedicle screw based on study of thoracic and lumbar pedicle of Indonesian population instead of 6.2 mm of diameter of commercially available pedicle screw. Our pedicle screws were made of AISI 316L and being produced at UPT Logam in Yogyakarta city of Indonesia by using Computerized Numerical Control (CNC) machines. The human resources consist of orthopaedic surgeon, resident and technician team. Statistical calculations were carried out with the IBM SPSS Statistics ver. 22.0 (IBM Co., Armonk, NY, USA) (Fig. 1).

We performed insertion time test, pull-out strength test and measurement of screw-media interface area for all groups of pedicle screws. Pull-out strength test was performed based on ASTM F543-02 as the Standard Specification and Test Methods for Metallic Medical Bone Screws. We embed the pedicle screw on the block of balsa wood. The head of pedicle screw was fixated by load fixture and the balsa wood was fixated by block holder on the load frame. Pull-out testing machine applied axial pulling force for ± 5 mm/minute till the pedicle screw was detached from the block. The tests were being performed at laboratory of mechanical engineering faculty of Universitas Gadjah Mada.

### Result

We evaluated 4 groups of pedicle screws that consist of 15 customized pedicle screws and 5 commercially available pedicle screws. Presented in Table 1, the mean of insertion time for group A was 22.94 s, 17.04 s for group B, 15.57 s for group C, and 15.86 s for group D. The mean of pull-out strength for group A was 746.6 N, 836.6 N for group B, 692.4 N for group C, and 408.6 N for group D. The measurement of screw-media interface area of group A was 1147.93 mm^2^, 1486.21 mm^2^ for group B, 1473.33 mm^2^ for group C, and 1054.63 mm^2^ for group D.

**Table 1:** Means of insertion time and pull-out strength

Descriptive result				
	N	Minimum	Maximum	Mean ± SD
Insertion time (s)				
V cylinder (A)	5	19.97	28.85	22.94 ± 1.54
Square conical (B)	5	15.79	18.88	17.04 ± 0.64
Square cylinder (C)	5	10.85	19.27	15.57 ± 1.56
Commercial (D)	5	9.19	22.72	15.86 ± 2.44
Total	20	9.19	28.85	17.85 ± 1.02
Pullout strength (N)				
V cylinder (A)	5	476	1043	746.60 ± 97.89
Square conical (B)	5	618	1143	836.60 ± 95.05
Square cylinder (C)	5	579	860	692.40 ± 48.54
Commercial (D)	5	314	541	408.60 ± 43.23
Total	20	314	1143	671.05 ± 50.50

The results of insertion time test show that group B pedicle screw with square-thread conical-core design has moderate insertion time compare to other pedicle screw groups. The results of pull-out strength test show that group B pedicle screw has the highest pull-out strength compare to other pedicle screws and it is also correlated with the measurement of screw-media interface area which show that group B has the largest results.

Based on Table 2, it is showed that the insertion time of customized pedicle screws were not statistically different compare to commercially available pedicle screw except for v-thread cylinder-core group which showed the slowest insertion time. It is also showed that the pull-out strength of group B pedicle screw was significantly higher than commercially available pedicle screw.

**Table 2:** Statistical analysis of insertion time

Dependent variable	Screw type	Mean difference	p value	95% CI
Insertion time	V Cylinder (A)			
	Square Conical (B)	5.90 ± 2.36	0.10	− 0.87–12.67
	Square Cylinder (C)	7.37* ± 2.36	*0.03*	0.56–14.13
	Commercial (D)	7.09* ± 2.36	*0.04*	0.31–13.86
	Square Conical (B)			
	V Cylinder (A)	− 5.90 ± 2.36	0.10	− 12.67–0.87
	Square Cylinder (C)	1.47 ± 2.36	0.92	− 5.30–8.24
	Commercial (D)	1.20 ± 2.36	0.95	− 5.60–7.96
	Square Cylinder (C)			
	V Cylinder (A)	− 7.37* ± 2.36	*0.03*	− 14.13–0.60
	Square Conical (B)	− 1.47 ± 2.36	0.92	− 8.24–5.30
	Commercial (D)	− 2.82 ± 2.36	0.99	− 7.05–6.50
	Commercial (D)			
	V Cylinder (A)	− 7.09* ± 2.36	*0.04*	− 13.85–0.31
	Square Conical (B)	− 1.19 ± 2.36	0.96	− 7.96–5.60
	Square Cylinder (C)	0.28 ± 2.36	0.99	− 6.50–7.05
	V Cylinder (A)			
Pull-out Strength	Square Conical (B)	− 90.00 ± 106.87	0.834	− 395.76–215.76
	Square Cylinder (C)	54.20 ± 106.87	0.956	− 251.56–359.96
	Commercial (D)	338.00* ± 106.87	0.028	32.24–643.76
	Square Conical (B)			
	V Cylinder (A)	90.00 ± 106.87	0.834	− 215.76–395.76
	Square Cylinder (C)	144.20 ± 106.87	0.547	− 161.56–449.96
	Commercial (D)	428.00* ± 106.87	0.005	122.24–733.76
	Square Cylinder (C)			
	V Cylinder (A)	− 54.20 ± 106.87	0.956	− 359.96–251.56
	Square Conical (B)	144.20 ± 106.87	0.547	− 449.96–161.56
	Commercial (D)	283.80 ± 106.87	0.074	− 21.96–589.56
	Commercial (D)			
	V Cylinder (A)	− 338.00* ± 106.87	0.028	− 643.76–32.24
	Square Conical (B)	− 428.00* ± 106.87	0.005	− 733.76–122.24
	Square Cylinder (C)	− 283.80 ± 106.87	0.074	− 589.56–21.96

*The mean difference is significant at 0.05 level. Significant value is identified with bold marks

### Discussion

This study found that the insertion time were significantly difference between v-thread cylinder-core pedicle screw (22.94 s) with commercially available pedicle screw (15.86 s) (p < 0.05). The pull-out strength was significantly difference between commercially available pedicle screw (408.60 N) with square-thread conical pedicle screw (836.60 N) (p < 0.05). The square-thread conical-core group have the highest interface area (1486.21 mm^2^).

The insertion time of pedicle screw is important because it can be a factor that influence how fast a surgeon can finish the operation. The longer the time taken to insert a pedicle screw means the longer the duration of surgery and the higher the risk of complications. Cheng Hang had reported that every additional of 15 min for surgery times can increase the infection rate by 13% [8].

Study by Higashino showed that pedicle screw removal was found to be 435.6 N in osteoporotic vertebra [9], which is comparable to this study for measurement of commercially available pedicle screw group (408.6 N). This study propose that best combination of pedicle screw design is square-thread conical-core design regarding the statistical analysis due to its comparable insertion time and higher pull-out strength compare to commercially available pedicle screw.

Screw loosening was found between 0.6 and 11% and might be higher in osteoporotic bone. Re operation rate because pedicle screw loosening was found between 14 and 27% [10]. Fixation failure because of screw loosening might change the spinal alignment and fixation stability that might harm the patient.

The geometry of the pedicle screw core can be conical, cylindrical or combination of both. The three types have different mechanical strengths. Abshire et al. compared the conical and cylindrical core designs and concluded that the conical screw has a better pull-out strength than the core cylinder [3]. However, Kwok et al. carried out a similar study and found that there was no significant difference in the pull-out strength between screw with conical design and cylinder core [11]. Meanwhile, Yaman et al. examined the pull-out strength of a pedicle screw with dual core and found that the design had a higher pull-out strength value compared to the screw with conical and cylinder cores [1]. The limitation in this study is that we cannot use a pedicle screw with dual cores due to the limited production equipment.

Kim et al. examined the shape of the thread on a screw and its effect on pull-out strength and found that threads with a v-thread design had a higher pull-out strength compared to square threads. According to their study, this is influenced by the flank overlap area (FOA) and thread pitch where the screw which has a larger flank overlap area and a smaller thread pitch has a higher pull-out strength value [4]. This is different from the results we obtained in this study where the pull-out strength value of a pedicle screw with a square thread has a higher pull-out strength value than a pedicle screw with a v-thread. This may be influenced by the contact surface area between the screw and the larger media on the pedicle screw with the square thread which in this study was successfully measured using the solid works software program. This difference certainly requires further study and further research with a larger sample size may be required.

### Conclusion

This study showed that our institution has been able to develop customized pedicle screws that the square-thread conical-core customized pedicle screw group has comparable insertion time and has better pull-out strength than commercially available pedicle screw.

## Limitation

Our recent development of pedicle screw has only designed for lumbar segment of vertebra. Further research is mandatory to develop another pedicle screw for thoracic and cervical segment. We propose the continuation of this research by comparing designed pedicle screw of lumbar, thoracic and cervical segment.

## Data Availability

This study doesn’t contain any individual data. The datasets used and/or analysed during the current study are available from the corresponding author on reasonable request. The data is available on this manuscript.
